# Systematic Retesting for *Helicobacter pylori*: The Potential Overestimation of Suppressive Conditions

**DOI:** 10.1155/2022/5380001

**Published:** 2022-04-25

**Authors:** Richard F. Knoop, Pauline C. Gaertner, Golo Petzold, Ahmad Amanzada, Volker Ellenrieder, Albrecht Neesse, Sebastian C. B. Bremer, Steffen Kunsch

**Affiliations:** ^1^Department of Gastroenterology, Gastrointestinal Oncology and Endocrinology, University Medical Center Göttingen, Georg-August University, Göttingen, Germany; ^2^Department of Gastroenterology, Internal Medicine and Geriatrics, Rems-Murr Hospital, Winnenden, Germany

## Abstract

**Background and Aims:**

In contrast to guideline recommendations, endoscopic testing for *Helicobacter pylori* is frequently performed under *Helicobacter pylori* suppressive conditions, e.g., intake of proton-pump inhibitors (PPI), preceded antibiotic treatment, or recent gastrointestinal bleeding. Our study's aim was to retest patients with—under suppressive conditions—negative test results. This was carried out in order to examine the rate of false negative tests previously gathered under suppressive conditions.

**Methods:**

The trial was conducted in a large patient collective in a university hospital. Every elective esophagogastroduodenoscopy from in- and outpatients was included. Prior to endoscopy, suppressive conditions were collected via standardized questionnaire. If *Helicobacter pylori* testing was indicated, both helicobacter urease test and histology were performed in analogy to the Sydney classification. In case of a negative result under suppressive conditions, the patient was reinvited after, if possible, withdrawal of suppressive condition in order to perform a urea breath test (UBT).

**Results:**

1,216 patients were included (median 59 years, 72.0% inpatients, 28.0% outpatients). Overall, 60.6% (737) were under *Helicobacter pylori* suppressive conditions. The main suppressive condition was intake of PPIs (54.5%). In 53.7% (653) of all included cases, *Helicobacter pylori* testing was performed. Of those, 14.1% (92) had a positive test, and 85.9% (561) were negative. Out of the patients with negative result, 50.8% (285) were tested under suppressive conditions and consequently invited for retesting via UBT. In 20.4% (45), suppressive conditions could not be ceased. In 22.8% (65), retesting was conducted. Of those, 98.5% (64) congruently presented a negative result again, and only 1.5% (1) was positive for *Helicobacter pylori*.

**Conclusion:**

Many patients undergoing esophagogastroduodenoscopy in everyday clinical practice are tested for *Helicobacter pylori* under suppressive conditions leading to a potentially higher risk of false negative results. However, our research shows that this issue might be overestimated.

## 1. Introduction

Over the last decades, the prevalence of infections with *Helicobacter pylori* (*H. pylori*) has decreased [1]. Nevertheless, about 50% of the adult world population aged over 40 years remains infected with a wide variety of prevalence not only between industrial and developing countries but also within a single population [2–5]. For instance, in Germany, *H. pylori* infection shows a low prevalence in children (3%) and ranges from 20 to 40% in adults [1, 6–8]. For immigrants, it is significantly higher (36-86%) [1, 9].

*H. pylori* infection induces an active chronic gastritis, possibly leading to dyspeptic syndromes, gastroduodenal ulcer disease, gastric cancer, or gastric mucosa-associated lymphoid tissue (MALT) lymphoma as well as extra-intestinal diseases [6, 10]. Yet, there are no sufficient prevention strategies. In particular, an effective vaccine has not been available so far [1].

The decision for *H. pylori* testing and the selection of the diagnostic test should follow the recommendations summarized in various guidelines [1, 11, 12]. *H. pylori* can be detected by several adequately validated tests [13]. The noninvasive assays include urea breath test (UBT), serologic immunoglobulin G (IgG) antibodies, and stool antigen test with monoclonal antibodies. UBT can be regarded as gold standard of noninvasive *H. pylori* tests with high sensitivity and specificity of both up to over 95% [14–16]. For UBT, the patient is administered urea labeled with a carbon isotope, usually the nonradioactive carbon-13 [14]. In the following 10 to 30 minutes, the isotope-labeled carbon dioxide in the patient's exhaled breath indicates the splitting of urea by *H. pylori*'*s* enzyme urease proving the presence of *H. pylori* [17, 18].

The invasive methods include helicobacter urease test (HUT), histology, culture, and polymerase chain reaction (PCR) from gastric biopsies [1, 14, 19]. The mentioned methods show different sensitivities and specificities. However, none is perfect in its accuracy [14, 20, 21]. HUT, e.g., reaches a sensitivity from 85 to 100% and a specificity up to 100% [13, 22]. Histology shows a sensitivity and specificity of both around 94% [13].

A major tool to diagnose *H. pylori* in everyday clinical practice is endoscopic biopsy. Principally, during every esophagogastroduodenoscopy (EGD), the decision whether to test or not to test for *H. pylori* should be made. If indicated, testing should be undertaken with biopsies for at least two different tests in accordance with the Sidney classification which primarily recommends histology and HUT [1, 23, 24].

Following the scientific literature, the sensitivity of all tests, apart from serology, is supposed to be impaired by conditions that lead to a reduced *H. pylori* colonization density [1, 14, 25, 26]. These are in particular the treatment with a proton-pump inhibitor (PPI), preceded *H. pylori* affecting antibiotic treatment and recent upper gastrointestinal bleedings [1, 27–29]. These confounding factors are a diagnostic challenge that can lead to a decreasing sensitivity and consequently to a higher rate of potentially false negative *H. pylori* test results [5]. Therefore, guidelines actually recommend a minimal interval of 2 weeks after completing a PPI therapy and 4 weeks after previous antibiotic therapy [1].

Nevertheless, *H. pylori* testing is practically often conducted under suppressive conditions despite the clear statements [5, 30]. Moreover, patients under *H. pylori* suppressive conditions being tested during EGD can even represent the majority; which could, e.g., be shown by previous work of our group [5]. For instance, this is due to many patients with dyspepsia already being primarily treated empirically with a PPI before an EGD with *H. pylori* testing can be conducted [1]. Obviously, suppressive conditions are a diagnostic “black box” of *H. pylori*. This might be clinically relevant for symptom control and long-term implications, e.g., gastric cancer incidence. Furthermore, patients being tested under suppressive conditions may even be disadvantaged as in clinical practice a negative test result is often stated “negative” regardless of potential *H. pylori* suppressive conditions which are often also not accurately assessed. Consequently, testing of *H. pylori* under suppressive conditions remains an unsolved clinical issue [5].

The guideline recommendations concerning *H. pylori* suppressive conditions are either based on partially old experimental laboratory data concerning *H. pylori* colonization density or on clinical data with inferior evidence, e.g., gained by logistic regression analysis [1, 27, 31–35]. To the best of our knowledge, there are no systematic clinical studies addressing this relevant question in present time.

That is why we performed a clinical trial with a high number of cases in order to systematically investigate the relevant and common clinical dilemma of potentially false negative *H. pylori* test results under suppressive conditions. The purpose of our study was to answer the question whether previous laboratory findings stating *H. pylori* suppressive conditions leading to false negative test results are reproducible in a real world setting or whether they might be overestimated in clinical practice.

## 2. Materials and Methods

This study was carried out in a large single center patient collective of the University Medical Center Göttingen, a tertiary German referral center. Our clinical standard of testing *H. pylori* followed the indications according to the German S2k-guideline [1].

All patients who underwent elective EGD were included. Prior to EGD, the indication for *H. pylori* testing was assessed and documented accurately following our clinical standard. If testing was indicated, always both HUT (Pronto Dry New, Gastrex, Gilly-lès-Cîteaux, France) and histology were conducted. HUTs were interpreted 1 hour after EGD according to the manufacturer's instructions. Data were collected over a period of 6 months. Inpatients as well as outpatients were included. EGDs were conducted by experienced endoscopists. As guidelines recommend and in analogy to the Sydney classification, biopsies were obtained from both the corpus (greater and lesser curvature) and antrum (greater and lesser curvature) [1, 23].

The study was approved by the local Institutional Ethics Committee (case 9/11/20) and conformed to the Helsinki Declaration as well as local legislation.

### 2.1. Data Collection

Following our clinical standard, the medical history of every patient was assessed routinely prior to elective EGD. This was recorded by standardized questionnaire with regard to the detection of *H. pylori* suppressive conditions.

For this, the following parameters were obtained: patient's age, sex, inpatient or outpatient, date of EGD, previous intake of PPI (within the last 2 weeks), previous antibiotic treatment (within the last 4 weeks), and signs of current upper gastrointestinal bleeding (hematemesis, melena within the last 3 days)

If invasive *H. pylori* testing was performed, the results of histology and HUT were recorded. Subsequently, *H. pylori* negative results (both histology and HUT) under suppressive conditions were detected. Following our clinical standard and the guidelines, concerned patients were invited telephonically for a UBT (INFAI, Köln, Germany) after—if possible—cessation of *H. pylori* suppressive conditions.

### 2.2. Statistical Analysis

Statistical analyses were performed with GraphPad Prism version 9.1.2 (GraphPad Software, San Diego, California, USA) and with SPSS Statistics version 26.0.0.0. (IBM, Armonk, NY, USA). Data were reported as mean including standard deviation. Differences between two groups were performed with two-tailed Mann–Whitney test or two-sided chi-square test.

*p* values less than 0.05 were considered statistically significant and are marked by ^∗^.

## 3. Results

### 3.1. Suppressive Conditions

**Figure 1:**
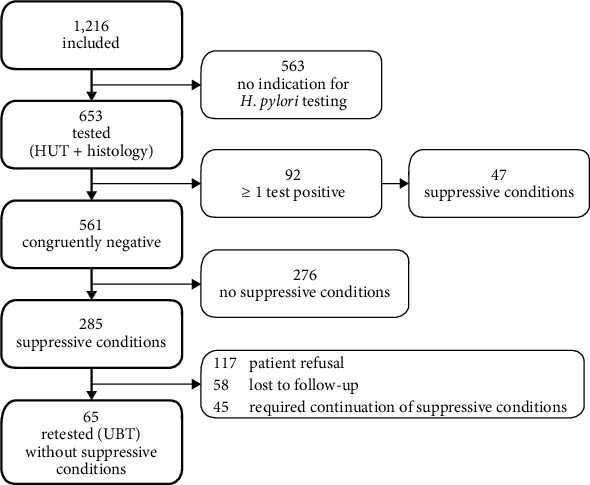
Study design.

The study included 1,216 patients (Figure 1) with a median age of 59 years. 340 (28.0%) were outpatients, and 876 (72.0%) were inpatients. Overall, 737 (60.6%) patients had one or more *H. pylori* suppressive conditions, whereas only 479 (39.4%) had no *H. pylori* suppressive conditions at all. PPI intake was the major suppressive condition in 54.5% (663/1,216) of all patients followed by antibiotic treatment within the previous 4 weeks with 17.0% (207/1,216) and clinical signs of recent upper gastrointestinal bleeding (hematemesis, melena) with 11.6% (141/1,216).

### 3.2. H. pylori Testing

653 (53.7%) of all included 1,216 patients (Figure 1) had the indication for *H. pylori* testing which was always conducted by both histology and HUT. Patients indicated for testing were characterized by a reduced percentage of *H. pylori* suppressive conditions (50.8% vs. 71.9%; *p* < .001^∗^), a lower median age (54 vs. 65 years; *p* < .001^∗^) and a higher percentage of outpatients (36.1% vs. 18.5%; *p* < .001^∗^) compared to nontested patients (Table 1).

The detailed analysis of suppressive conditions between the tested and nontested patients showed a reduced intake of PPIs (45.2% vs. 65.4%; *p* < .001^∗^), reduced intake of antibiotics (13.0% vs. 21.7%; *p* < .001^∗^), and a lower rate of upper GI-bleedings (6.4% vs. 17.6%; *p* < .001^∗^) among the tested individuals (Table 1).

Of the 653 tested patients, 92 (14.1%) had a positive test for *H. pylori* whereas 561 (85.9%) were negative for both histology and HUT (Figure 1).

These two subgroups (tested positive vs. tested negative) were similar concerning suppressive conditions (51.1% vs. 50.8%; *p* ≥ .999; Table 2). Furthermore, no significant differences concerning age (51.5 vs. 55.0 years; *p* = .389), sex (*p* = .653), and rate of outpatients (37.0% vs. 36.0%; *p* = .907) were observed (Table 2).

Of the 92 *H. pylori* positive patients, 41 (44.6%) showed an incongruent result (one test positive, one test negative). 35 (85.4%) of the incongruent results appeared under *H. pylori* suppressive conditions, mainly under PPI (27/35; 77.1%).

In sum, 285 out of the 561 patients (50.8%) with negative results were investigated under suppressive conditions (Figure 1, Table 2) coming along with a potentially increased risk of a false negative test results.

### 3.3. Retesting for H. pylori via UBT after Withdrawal of Suppressive Conditions

Following the clinical standard of our hospital as well as the guidelines, all of those 285 patients tested negatively under suppressive conditions were offered an outpatient appointment in order to conduct a urea breath test (UBT) with ceased *H. pylori* suppressive conditions (Figure 1).

In 65 patients (22.8%), a UBT was performed after withdrawal of *H. pylori* suppressive conditions for at least 4 weeks. Interestingly, only one of these patients (1.5%) presented an *H. pylori* positive result in the UBT. Previously, this single patient had been treated with a PPI as suppressive condition. All other 64 UBTs (98.5%) again tested negatively.

Despite active informing of the patients, in 220 cases (77.2%), no UBT could be performed (Figure 1). The clustered reasons for this were “suppressive conditions cannot be ceased” with 20.5% (*N* = 45), “patient refusal” 53.2% (*N* = 117), and “lost to follow-up” 26.4% (*N* = 58) (Figure 1).

## 4. Discussion

This study addresses the challenge of a high rate of *H. pylori* tests being conducted under *H. pylori* suppressive conditions. We focused on this relevant clinical issue because it is suspected to lead to more false negative test results. That is why we retested those patients with initially negative *H. pylori* test results under *H. pylori* suppressive conditions after withdrawal of the suppressive condition by performing a urea breath test (UBT).

Following the scientific literature, the main *H. pylori* suppressive conditions are treatment with PPIs, recent antibiotic treatment and upper gastrointestinal bleeding leading to a reduced sensitivity of all common *H. pylori* tests due to a reduction of *H. pylori* colonization density [1, 14, 25–29]. Therefore, guidelines obviously recommend testing under nonsuppressive conditions [1]. However, this does not always meet the clinical practice [5]. In particular, the withdrawal of PPIs can often not be realized [1]. For instance, many patients with dyspepsia have already been treated with PPIs before an EGD can be performed and *H. pylori* is tested [1].

Our cohort shows a real word setting of *H. pylori* suppressive conditions in patients undergoing EGD in a German university hospital. The data demonstrate that even the majority of patients show *H. pylori* suppressive conditions, leading to the relevant diagnostic challenge of how to deal with their negative *H. pylori* test result.

Throughout the trial, a large absolute and relative amount of negatively tested individuals exhibited *H. pylori* suppressive conditions (285 out of 561 negatively tested patients; 50.8%). Interestingly and relevant in terms of internal validity of the study, the two subgroups of positively tested vs. negatively tested patients were similar concerning suppressive conditions (51.1% vs. 50.8%; *p* > .999).

In the following, the negatively tested patients under *H. pylori* suppressive conditions were the subgroup we put our focus on in order to answer the question whether they had previously shown a higher rate of false negative *H. pylori* test results. Following our clinical standard and the current guideline, all of those 285 patients were actively reinvited in order to undergo a urea breath test after withdrawal of *H. pylori* suppressive conditions. However, in many of those patients (20.5%), the *H. pylori* suppressive conditions could not be ceased. Furthermore, many of those patients do not suffer from symptoms anymore and are consequently not necessarily interested in an additional test. Therefore, it can be regarded as a success, that in 65 patients (22.8%), a UBT was eventually performed after cessation of *H. pylori* suppressive conditions. The most important result of our study is that only one patient (1.5%) then presented an *H. pylori* positive result in the UBT, whereas all other 64 UBTs (98.5%) were tested negatively again.

Obviously, there are certain limitations of our trial, e.g., concerning geographical differences of *H. pylori* prevalence. The monocentric study was conducted in a large German university hospital providing general and maximum care with a distinct patient collective that does not necessarily represent the general population. As a matter of course, patients with initially negative *H. pylori* test results under suppressive conditions who required continuation of particularly PPI therapy could not be retested without suppressive conditions. Apparently, the acceptancy rate of performed UBT seems to be low. However, the number of cases with necessarily continued PPI therapy as well as the fact that many patients do not show up for another test despite active telephonic invitation can themselves be considered as relevant results of the trial.

## 5. Conclusion

Our real-world data show that the sensitivity of testing *H. pylori* under suppressive conditions might not be as low as always suspected. This is of high clinical relevance because it could relevantly simplify testing for *H. pylori*.

## Figures and Tables

**Table 1:** Overall patients' characteristics.

Characteristic	Tested for *H. pylori* (HUT + histology) *n* = 653	Not tested due to missing indication = 563	*p*
Suppressive conditions (no. (%))	332 (50.8)	405 (71.9)	< .001^∗^
Age median (years)	54.0	65.0	< .001^∗^
Sex female (no. (%))	342 (52.4)	241 (42.8)	.001^∗^
Outpatients (no. (%))	236 (36.1)	104 (18.5)	< .001^∗^
PPI intake (no. (%))	295 (45.2)	368 (65.4)	< .001^∗^
Antibiotics intake (no. (%))	85 (13.0)	122 (21.7)	< .001^∗^
GI bleeding (no. (%))	42 (6.4)	99 (17.6)	< .001^∗^

*H. pylori: Helicobacter pylori*; HUT: Helicobacter urease test; GI: gastrointestinal; PPI: proton-pump inhibitor.

**Table 2:** Characteristics of tested patients.

Characteristic	Tested positive for *H. pylori* (HUT and/or histology) *n* = 92	Tested negative for *H. pylori* (HUT and histology) *n* = 561	*p*
Suppressive conditions (no. (%))	47 (51.1)	285 (50.8)	> .999
Age median (years)	51.5	55	.389
Sex female (no. (%))	46 (50.0)	296 (52.8)	.653
Outpatients (no. (%))	34 (37.0)	202 (36.0)	.907
PPI intake (no. (%))	38 (41.3)	257 (45.8)	.432
Antibiotics intake (no. (%))	15 (16.3)	70 (12.5)	.317
GI bleeding (no. (%))	11 (12.0)	31 (5.5)	< .035^∗^

*H. pylori: Helicobacter pylori*; HUT: Helicobacter urease test; GI: gastrointestinal, PPI: proton-pump inhibitor.

## Data Availability

Data available on request.

## Abbreviations

EGD: Esophagogastroduodenoscopy
H. pylori: Helicobacter pylori
IgG: Immunoglobulin G
PPI: Proton-pump inhibitor
HUT: Helicobacter urease test
n.s.: Not significant
UBT: Urea breath test

## References

[1] Fischbach W., Malfertheiner P., Lynen Jansen P. (2016). S2k-guideline Helicobacter pylori and gastroduodenal ulcer disease. *Zeitschrift für Gastroenterologie*.

[2] Peleteiro B., Bastos A., Ferro A., Lunet N. (2014). Prevalence of Helicobacter pylori infection worldwide: a systematic review of studies with national coverage. *Digestive Diseases and Sciences*.

[3] Malfertheiner P., Link A., Selgrad M. (2014). *Helicobacter pylori*: perspectives and time trends. *Nature Reviews. Gastroenterology & Hepatology*.

[4] Malfertheiner P., Chan F. K., McColl K. E. (2009). Peptic ulcer disease. *Lancet*.

[5] Knoop R. F., Petzold G., Amanzada A. (2020). Testing of *Helicobacter pylori* by endoscopic biopsy: the clinical dilemma of suppressive conditions. *Digestion*.

[6] Fischbach W., Malfertheiner P. (2018). Helicobacter Pylori Infection. *Deutsches Ärzteblatt Internationa*.

[7] Wex T., Venerito M., Kreutzer J., Götze T., Kandulski A., Malfertheiner P. (2011). Serological prevalence of *Helicobacter pylori* infection in Saxony-Anhalt, Germany, in 2010. *Clinical and Vaccine Immunology*.

[8] Michel A., Pawlita M., Boeing H., Gissmann L., Waterboer T. (2014). *Helicobacter pylori* antibody patterns in Germany: a cross-sectional population study. *Gut pathogens*.

[9] Porsch-Ozcürümez M., Doppl W., Hardt P. D. (2003). Impact of migration on Helicobacter pylori seroprevalence in the offspring of Turkish immigrants in Germany. *The Turkish Journal of Pediatrics*.

[10] Marshall B. J., Warren J. R. (1984). Unidentified curved bacilli in the stomach of patients with gastritis and peptic ulceration. *Lancet*.

[11] Chey W. D., Leontiadis G. I., Howden C. W., Moss S. F. (2017). ACG clinical guideline: treatment of helicobacter pylori infection. *The American Journal of Gastroenterology*.

[12] Malfertheiner P., Megraud F., O'Morain C. A. (2017). Management of *Helicobacter pylori* infection-the Maastricht V/florence consensus report. *Gut*.

[13] Abadi T. B. (2018). Diagnosis of *helicobacter pylori* using invasive and noninvasive approaches. *Journal of pathogens*.

[14] Sabbagh P., Mohammadnia-Afrouzi M., Javanian M. (2019). Diagnostic methods for Helicobacter pylori infection: ideals, options, and limitations. *European Journal of Clinical Microbiology & Infectious Diseases*.

[15] Wang Y. K., Kuo F. C., Liu C. J. (2015). Diagnosis of Helicobacter pylori infection: current options and developments. *World Journal of Gastroenterology*.

[16] Leal Y. A., Flores L. L., Fuentes‐Pananá E. M., Cedillo‐Rivera R., Torres J. (2011). 13C-urea breath test for the diagnosis of *Helicobacter pylori* infection in children: a systematic review and meta-analysis. *Helicobacter*.

[17] Levine A., Shevah O., Miloh T. (2004). Validation of a novel real time ^13^C urea breath test for rapid evaluation of *Helicobacter pylori* in children and adolescents. *The Journal of Pediatrics*.

[18] Shirin H., Kenet G., Shevah O. (2001). Evaluation of a novel continuous real time (13)C urea breath analyser for Helicobacter pylori. *Alimentary Pharmacology & Therapeutics*.

[19] Marshall B. J., Warren J. R., Francis G. J., Langton S. R., Goodwin C. S., Blincow E. D. (1987). Rapid urease test in the management of campylobacter pyloridis-associated gastritis. *The American Journal of Gastroenterology*.

[20] Cutler A. F., Havstad S., Ma C. K., Blaser M. J., Perez-Perez G. I., Schubert T. T. (1995). Accuracy of invasive and noninvasive tests to diagnose *Helicobacter pylori* infection. *Gastroenterology*.

[21] Best L. M., Takwoingi Y., Siddique S. (2018). Non-invasive diagnostic tests for *Helicobacter pylori* infection. *Cochrane Database of Systematic Reviews*.

[22] Bezmin Abadi A. T., Taghvaei T., Wolfram L. (2011). Inefficiency of rapid urease test for confirmation of *Helicobacter pylori*. *Saudi Journal of Gastroenterology*.

[23] Dixon M. F., Genta R. M., Yardley J. H., Correa P. (1996). Classification and grading of gastritis. *The American Journal of Surgical Pathology*.

[24] Fischbach W., Malfertheiner P., Hoffmann J. C. (2009). S3-guideline "helicobacter pylori and gastroduodenal ulcer disease" of the German society for digestive and metabolic diseases (DGVS) in cooperation with the German society for hygiene and microbiology, society for pediatric gastroenterology and nutrition e. V., German society for rheumatology, AWMF-registration-no. 021 / 001. *Zeitschrift für Gastroenterologie*.

[25] Tytgat G. N. J. (1997). Antimicrobial Therapy for Helicobacter pylori Infection. *Gut*.

[26] van Leeuwen P. (2020). Choosing wisely: previously published gastroenterological recommendations of the "Klug entscheiden"-initiative. *Zeitschrift für Gastroenterologie*.

[27] Mégraud F., Lehours P. (2007). *Helicobacter pylori* detection and antimicrobial susceptibility testing. *Clinical Microbiology Reviews*.

[28] Gisbert J. P., Abraira V. (2006). Accuracy of *helicobacter pylori* diagnostic tests in patients with bleeding peptic ulcer: a systematic review and meta-analysis. *The American Journal of Gastroenterology*.

[29] Sanchez-Delgado J., Gene E., Suarez D. (2011). Has H. pylori prevalence in bleeding peptic ulcer been underestimated? A meta-regression. *The American Journal of Gastroenterology*.

[30] Shirin D., Matalon S., Avidan B., Broide E., Shirin H. (2016). Real-world *helicobacter pylori* diagnosis in patients referred for esophagoduodenoscopy: the gap between guidelines and clinical practice. *United European Gastroenterology Journal*.

[31] Patel S. K., Pratap C. B., Jain A. K., Gulati A. K., Nath G. (2014). Diagnosis of *Helicobacter pylori*: what should be the gold standard?. *World Journal of Gastroenterology*.

[32] Lerang F., Moum B., Mowinckel P. (1998). Accuracy of seven different tests for the diagnosis of Helicobacter pylori infection and the impact of H2-receptor antagonists on test results. *Scandinavian Journal of Gastroenterology*.

[33] Megraud F., Trimoulet pascale, Lamouliatte H., Boyanova L. (1991). Bactericidal effect of amoxicillin on Helicobacter pylori in an in vitro model using epithelial cells. *Antimicrobial Agents and Chemotherapy*.

[34] McColl K. E. (2010). Helicobacter pyloriinfection. *The New England Journal of Medicine*.

[35] Siavoshi F., Saniee P., Khalili-Samani S. (2015). Evaluation of methods for *H*. *pylori* detection in PPI consumption using culture, rapid urease test and smear examination. *Annals of Translational Medicine*.

